# Environmental particulate matter—one of the culprits in the development of caries

**DOI:** 10.3389/fpubh.2025.1559384

**Published:** 2025-04-16

**Authors:** Wenxin Du, Ruxia Hou, Xixi Li, Jiajia Liu, Tingting Yang, Junming Li, Junyu Liu, Xiangyu Wang

**Affiliations:** ^1^Department of Pediatric and Preventive Dentistry, School and Hospital of Stomatology, Shanxi Medical University, Taiyuan, China; ^2^Shanxi Province Key Laboratory of Oral Diseases Prevention and New Materials, Taiyuan, China; ^3^School of Statistics, Shanxi University of Finance and Economics, Taiyuan, China

**Keywords:** environmental particulate matter, dental caries, oral microbiota, immune system, oxidative stress, saliva

## Abstract

With the development of society, ecological and environmental problems have gradually become the focus of attention of countries around the world, among which environmental particulate matter poses a major harm to health. This article elucidates the association between environmental particulate matter and dental caries and provides new insights into the underlying mechanisms. In addition, this study emphasizes the role of oxidative stress in the occurrence and development of dental caries, and a new research pathway based on the interaction between oxidative stress and dental caries based on the Nrf2 pathway has become the focus of future research on the pathogenesis of dental caries. The relevant content of this review can provide a certain theoretical basis for the follow-up multidisciplinary joint research of researchers, and provide a certain reference for public health personnel and policymakers to formulate prevention strategies and public health interventions, carry out more accurate individualized treatment for high-risk groups, implement key prevention and treatment, and promote the overall improvement of effective prevention and treatment of caries. Ultimately, more attention must be paid to addressing the relationship between environmental particulate pollution and dental caries, with a focus on pollution control and reducing preventable environmental risks in order to protect oral health more broadly.

## 1 Introduction

In recent years, the acceleration of industrialization and urbanization has brought attention to air quality issues. The World Health Organization (WHO) has identified air pollution as a significant environmental health risk. As early as the 1950s, it was recognized that air pollution is associated with respiratory and cardiovascular diseases, leading to a relative increase in mortality and morbidity (1). In 2017, outdoor air pollution containing particulate matter was classified as a Class 1 carcinogen by the World Health Organization. Particulate matter (PM), including fine particulate matter (PM_2.5_) and inhalable particulate matter (PM_10_), is a major component of air pollution (2, 3).

**Figure 1:**
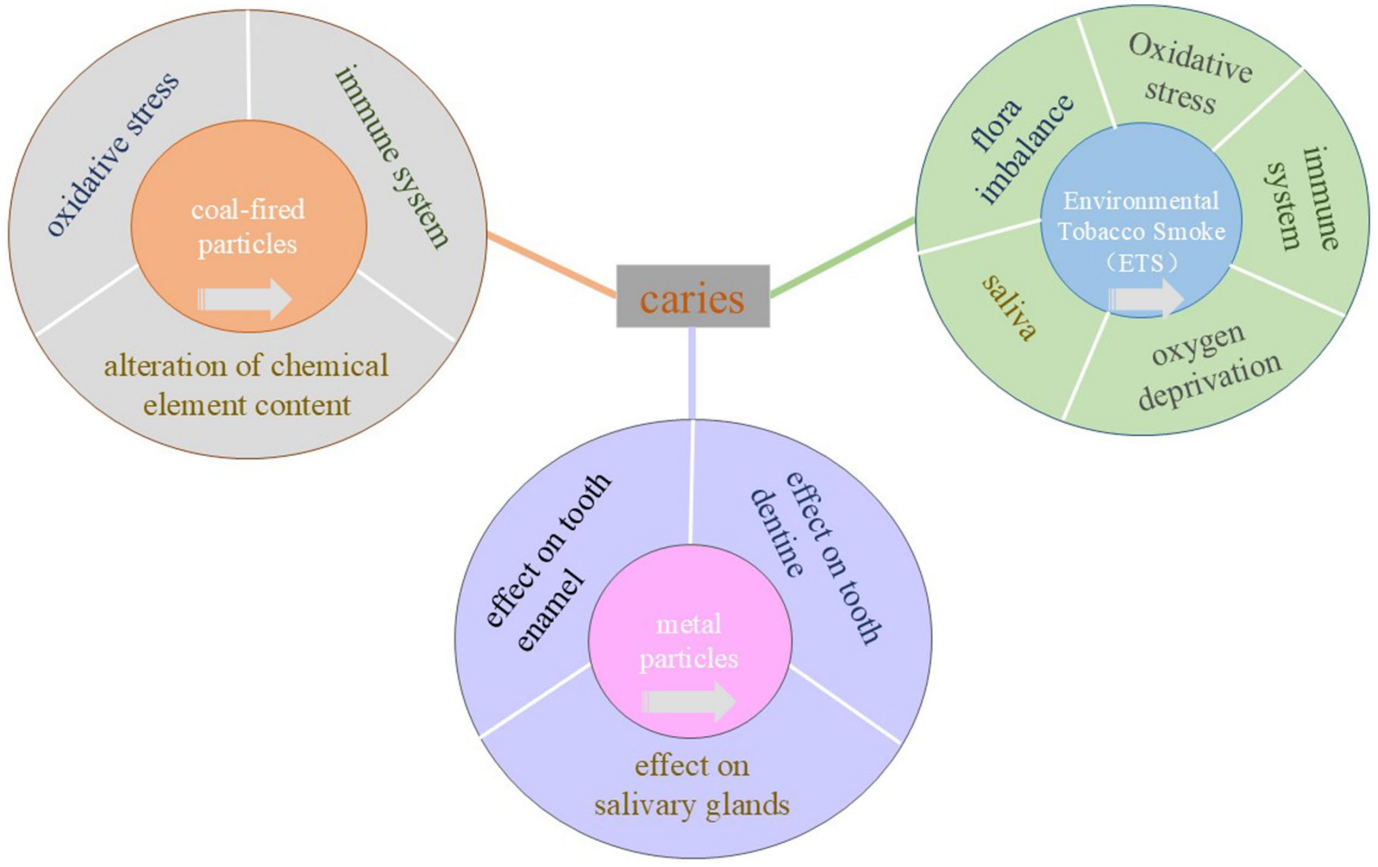
Specific mechanisms of caries induced by environmental tobacco smoke, coal-fired particles and lead and cadmium. The central rectangle represents caries, the inner layer of three concentric circles represents the three exposure factors, and the outer layer represents the pathological mechanism that causes caries. Click the arrow in the center circle to jump to that location.

Currently, environmental particulate matter is classified into various categories, including soil particles from natural sources, coal soot from man-made sources, and substances like arsenic, cadmium, chromium, nickel, emitted through incineration. Additionally, lead from automobile exhaust and particulate matter formed by the condensation of organic matter, such as alkanes, olefins, aromatic hydrocarbons, etc., are also considered. Numerous studies have demonstrated the significant impact of environmental particulate matter on various aspects of human health, including immunoglobulin levels, respiratory flora colonization, chromosome rupture in human blood lymphocytes, oxidative damage to vascular and epithelial cells, as well as increased prevalence of respiratory diseases and symptoms in children (4, 5). As the first gateway of the respiratory and digestive systems, the oral cavity is exposed to environmental particulate matter for a long time, which will have a certain impact on oral diseases. Long-term exposure to environmental particulate matter increases the risk of caries in children, depending on the age group. This is due to the fact that children’s immune system is not yet fully developed, and the harmful substances in PM can easily enter the body through the respiratory tract, interfere with the immune system, reduce the resistance of the oral mucosa, and make it easier for bacteria in the oral cavity to breed and multiply, thereby increasing the risk of dental caries. In addition, in children who are in the stage of growth and development, the process of mineralization of their teeth is not yet complete. Long-term exposure to PM contamination can interfere with the normal mineralization of teeth, leading to tooth dysplasia and thus increasing the risk of caries. For adults, the effect of environmental particulate matter on caries is mainly due to its alteration of the microbial community structure in the oral cavity, resulting in a decrease in beneficial bacteria and an increase in cariogenic bacteria. This dysbiosis can disrupt the ecological balance in the mouth and trigger tooth decay. Recent studies demonstrate that PM exposure influences oral health by disrupting microbiota balance and increasing oxidative stress (6). Current research is limited, and it is unclear whether there is an underlying mechanism. In this paper, we explore the potential relationship between environmental particulate matter and oral caries based on three main sources of environmental particulate matter, including environmental tobacco smoke, coal-fired particles, and heavy metal particles (Figure 1). No direct causal evidence links particulate matter (PM) to dental caries, but PM may indirectly contribute by altering the oral microenvironment. The analysis in this paper is not only limited to the correlation between environmental particulate matter and dental caries, but also explores the influencing mechanisms between the two, such as microbiota, saliva, oxidative stress, and other underlying factors. By summarizing the association between environmental particulate matter exposure and dental caries, this review provides new insights into the underlying mechanisms, in order to provide some direction for subsequent related research.

The literature selected for this review is up to 2025 and has collected published articles on the relationship between environmental particulate matter and dental caries prevalence from four search engines: PubMed, Web of science, Google Scholar, and China’s National Knowledge Infrastructure (CNKI). Then, according to the severity of environmental pollution, three kinds of particulate matter, such as tobacco smoke, coal burning particles and metal particles, which are harmful to the human body and have a significant impact on the occurrence of dental caries, are selected. Factors such as microbiota, host, etc., including dysbiosis, impaired immune system, salivary changes, and oxidative stress, were screened out from the 4 factors of dental caries.

## 2 Environmental tobacco smoke

**Figure 2:**
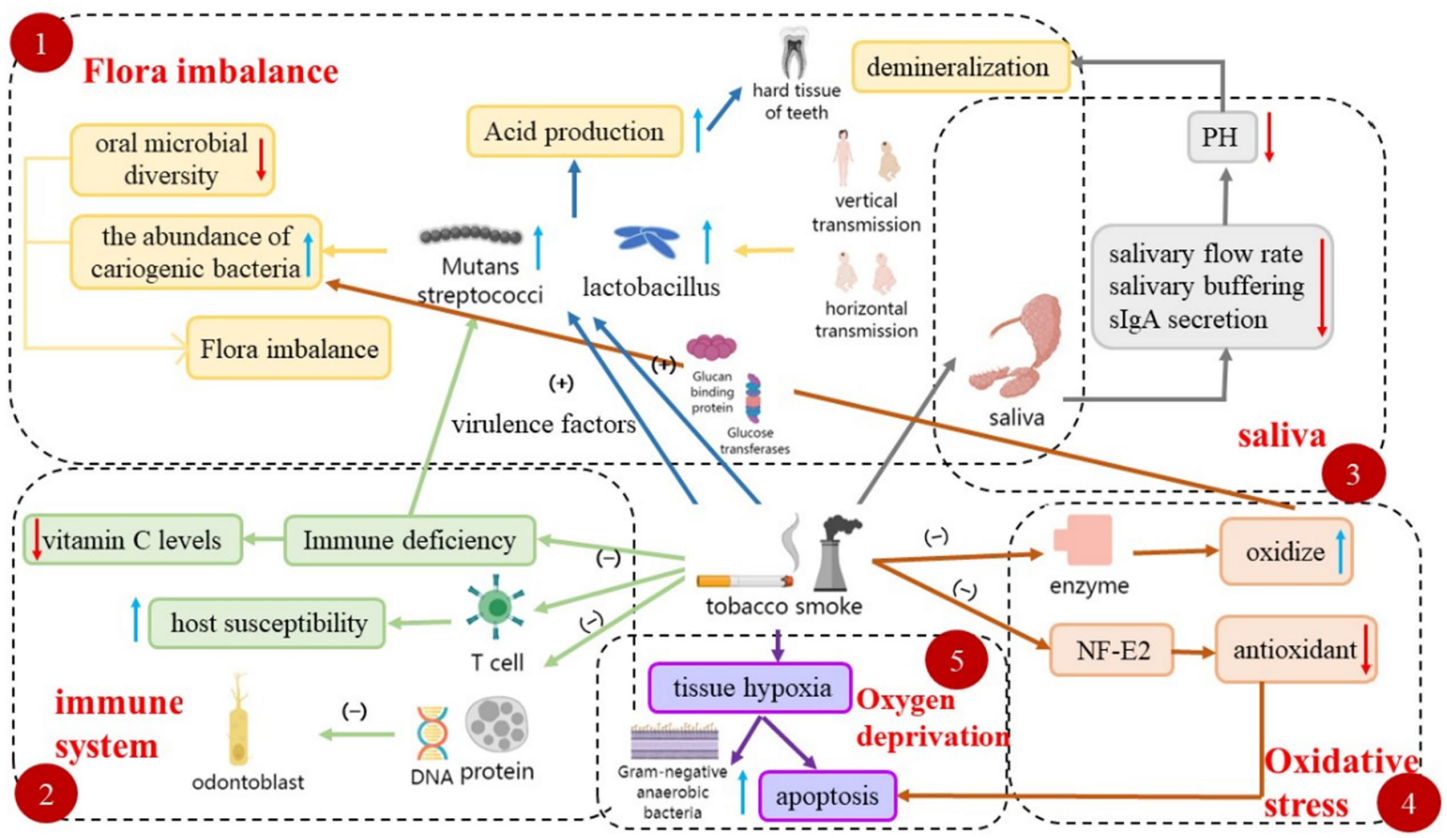
Multiple mechanisms involved in tobacco smoke-induced caries. This figure illustrates the diverse pathways involved in the development of tobacco smoke-induced caries, including dysbiosis, effects on the immune system, saliva, oxidative stress, and tissue hypoxia.

Dental caries remains a chronic, progressive, and devastating global health challenge, affecting nearly one-third of the global population, with a prevalence rate of 29% (7). Environmental tobacco smoke (ETS), also known as secondhand smoke, refers to tobacco smoke produced by smokers when they smoke. According to the literature, ETS is one of the main sources of indoor air pollutants and there is a strong relationship between it and caries. In a cross-sectional study in Pakistan, after adjusting for confounders, the association between environmental tobacco smoke exposure and dental caries was statistically significant (8). The study demonstrated an increased prevalence of dental caries in people exposed to tobacco smoke and demonstrated a distinct dose-dependent effect in a follow-up study of 405 participants (9). According to data from the Korean Adolescent Risk Behavior Network Survey, ETS exposure was positively associated with oral symptoms compared to adolescents without ETS exposure (10). However, one study showed an increase in dental caries experience with prenatal passive smoking compared to postnatal exposure (11). This should lead us to focus more on prenatal exposure in school-age children, with a cross-sectional analysis of school-age children showing a significant 41% increase in the prevalence of dental caries when children were exposed to ETS prenatally (12). Moreover, environmental tobacco smoke and early childhood caries have been bidirectionally validated in a study to identify key windows of ETS exposure associated with an increased risk of developing dental caries in children (13). The degree of prenatal exposure in school-age children may be more likely to affect the prevalence of dental caries after birth. However, the specific mechanism by which environmental tobacco smoke affects caries is not well understood. Current research evidence suggests that ETS may directly affect the composition of oral microbes, disrupt the balance of oral microecosystems, and lead to a decrease in biodiversity and an increase in cariogenic bacteria (14). It is also possible to alter the oral microenvironment, leading to changes in saliva flow rate, buffering capacity, and composition, which can contribute to the development of caries (15). In addition to local factors, tobacco smoke can affect systemic factors to cause caries. For example, ETS may disrupt the balance between oxidative and antioxidant systems in the body, exacerbate oral inflammation, promote apoptosis, and consequently facilitate the development of dental caries (16, 17). Additionally, ETS exposure is associated with early immune impairment, leading to immunodeficiency (18, 19), increasing host susceptibility and indirectly increasing the risk of dental caries (Figure 2).

### 2.1 Flora imbalance

The oral cavity hosts one of the most complex and diverse microbial communities in the human body (20–22). To date, approximately 700 species of oral bacteria have been identified, with the oral microbiota of a typical individual ccomprising 296 species. The balance in the number of these microbiota sustains the oral ecosystem’s stability (23). Whereas, dental caries arises from the collective activity of various microorganisms, primarily bacteria, due to an imbalance in oral microecology and changes in the microenvironment. The “ecological plaque hypothesis” suggests that dental caries result from the activity of heterogeneous microbial mixtures and cariogenic shifts within the plaque microbiome (24). In a five-year longitudinal study of salivary bacteria involving 189 children, researchers observed that bacterial aggregation could either promote or inhibit the development of dominant caries (25). These results suggest that changes in microbiota can cause changes in the prevalence of caries. Studies have shown that particles in tobacco smoke primarily disrupt the oral microbiota by decreasing microbial diversity and enhancing the abundance of cariogenic bacteria. Duan et al. (26) and Tsigarida et al. (27) utilized 16S ribosomal RNA gene sequencing and 454-pyrophosphate sequencing, respectively, and found that the microbial diversity of smokers decreased, while the number of some specific bacteria increased. For example, exposure to ETS resulted in a significant increase in the levels of firmicutes and actinomycetes at the phylum level (28). These findings align with earlier results showing that exposure to tobacco smoke enhances the presence of cariogenic bacteria.

Recent studies demonstrate that PM exposure influences oral health by disrupting microbiota balance. Long-term exposure to tobacco particulate matter may induce profound alterations in the microbiota, resulting in the loss of beneficial oral species, disruption of the or an ecosystem’s delicate balance, fostering the growth of cariogenic microorganisms (14), and ultimately advancing caries progression (29). The imbalance between cariogenic and commensal bacteria increases acid production, which erodes the tooth’s hard tissues and leads to caries. At present, most of the studies on long-term exposure to tobacco particulate matter causing microbiota imbalance and caries are observational studies, and it is difficult to determine its direct causal relationship, and the oral microbiota is easily affected by a variety of factors, such as oral hygiene, diet and other factors, and tobacco particulate matter as a secondary factor, its impact may be masked by other factors, therefore, more high-quality studies are needed in the future to clarify the relationship between tobacco particulate matter and oral microbiota and dental caries, and consider the influence of multiple confounding factors.

#### 2.1.1 Mutans streptococci

According to reports, *mutans streptococci* play a central role in the initiation of caries (30). Babies acquire *Streptococcus mutans* from their mothers mainly through vertical transmission. However, recent studies suggest that *Streptococcus mutans* can colonize infants’ mouths earlier before tooth eruption and horizontal transmission (among children) is possible (31). Early acquisition of *S. mutans* is associated with early childhood caries and increased risk of future caries, while delayed colonization is linked to reduced dental caries (32, 33). The effect of *Streptococcus mutans* on the prevalence of dental caries is not limited to early colonization, but is also affected by its level. In several studies, a positive association between salivary levels of *mutans streptococci* and the presence, prevalence, or incidence of carious lesions on different tooth surfaces (34–37). Tobacco smoke contains more than 7,000 compounds, such as nicotine, among others, which have been shown to promote the growth of *Streptococcus mutans*. The mechanism is that the pathogenicity of this bacterium is enhanced by glucosyltransferase (GTF). Nicotine in tobacco smoke can up-regulate the expression of glucan-binding protein (GBP) and GTF genes in planktonic *Streptococcus mutans*, and down-regulate their expression in biofilm communities (38), facilitating the bacterium’s adherence and accumulation on tooth surfaces and subsequent acid production, leading to tooth demineralization and caries formation. In addition, nicotine also enhances the expression of virulence factors such as non-woolenomycete mutant C (nlmC), lactate dehydrogenase (ldh), and phosphotransferase system-related genes (pts), increasing their cariogenic activity (39). In view of the competition and symbiosis between bacteria, the coexistence and competition between *Streptococcus mutans* and *Streptococcus surius* will increase the risk of caries and accelerate the development of dental caries to a certain extent. Cigarette smoking, on the other hand, as a long-term exposure factor, exacerbates the interaction between the two bacteria and promotes the development of dental caries (40, 41). The interaction between *S. sanguinis* and *S. mutans* is considered a significant factor in caries development (42). *S. sanguinis* is believed to be beneficial in inhibiting dental caries. On one hand, *S. sanguinis* and *S. mutans* compete for the same ecological niche and exhibit similar metabolic characteristics (43). On the other hand, *S. pneumoniae* can produce hydrogen peroxide and heme, which inhibit *S. mutans* (44). Nicotine in tobacco can enhance the competitive advantage of *S. mutans* over *S. sanguinis*, promoting caries development. The findings of Li et al. support this conclusion (40).

Not only that, but the transmission of *Streptococcus mutans* by a smoking mother to her child may be a significant factor in tooth decay in children. Epidemiological evidence shows that children of non-smoking mothers during pregnancy exhibit lower rates of dental caries compared to those whose mothers smoked during pregnancy. However, there is no significant increase in dental caries prevalence among children of mothers who quit smoking during pregnancy (45). It is noteworthy that individuals with higher levels of *Streptococcus mutans* in their oral cavity often have smoking habits, which may indicate poor oral hygiene, high life stress, neglect of health behaviors, and a higher susceptibility to dental caries.

#### 2.1.2 Lactobacilli

*Lactobacilli* represent another significant contributing species to tooth caries. Although they are late colonizers and not necessary for the onset of caries, they can significantly impact the progression of established lesions (46). A study by Avşar et al. (47) found that the colonization of *lactobacilli* in the saliva of children exposed to passive smoke was substantially higher than in the control group (*p* < 0.05), likely due to the nicotine in tobacco smoke promoting *lactobacilli* colonization. The mechanism lies in the fact that heavy metals such as arsenic, lead, and cadmium, present in smoke, can accumulate in the oral cavity, disrupting the acid–base balance and accelerating the activity of pathogenic bacteria.

#### 2.1.3 Other microorganisms

In addition to *streptococcus*, *actinomyces* are also considered cariogenic bacteria and contribute to the onset of root surface caries (48). They can survive and produce acid in environments below pH 5.5, the critical concentration for demineralization (49). Therefore, actinomycetes play a significant role in dental caries development. However, the effects of tobacco smoke on *actinomycetes* are controversial and further research is needed (50, 51).

In addition, *Candida albicans*, as a fungus, can cause dental caries, especially in children, adolescents, and young adults (52–55). This is due to its acidogenicity, the ability to form hyphae, and the secretion of dentine-degrading enzymes (56, 57). Early colonization of Candida has been reported to be associated with the onset of caries in deciduous molars and can be an important fungus that causes caries in deciduous teeth (58).

### 2.2 The immune system

Caries is a bacterial infection that is associated with changes in the immune system. In particular, immunoglobulin A (sIgA) in saliva plays an important role in the occurrence and development of dental caries. sIgA not only mediates the humoral immune response to regulate caries activity, but also interferes with the formation of caries, resulting in microbial adhesion to tooth surfaces and biofilms. Some studies have shown that microorganisms can protect themselves from host immune attack by forming biofilms and reducing antigen expression, which results in higher levels of sIgA in saliva in healthy people than in caries patients. But the activation pattern of sIgA in saliva still needs to be further studied. ETS can negatively affect the immune system through a variety of mechanisms, altering the body’s immune response to bacteria and viruses, increasing host susceptibility and increasing the risk of caries.

#### 2.2.1 Immune system deficiencies

Research has shown that there is growing concern about the role of immune system risk in the development of dental caries (59). Immunoglobulin (Ig), when bound to cariogenic surface antigens, prevents bacteria from adhering to the tooth surface. Tobacco smoke has been shown to impair the immune status of saliva (60), resulting in a decrease in immunoglobulin levels, which may be a moderate secondary immunodeficiency (61, 62). When IgA levels are low, cariogenic bacteria are more likely to adhere to and colonize the teeth, forming plaque, which in turn can lead to tooth decay. In addition, IgA neutralizes toxins and enzymes produced by cariogenic bacteria, such as glycosyltransferases (GTFs), which promote the formation of biofilms on the tooth surface. When IgA is lowered, the action of these enzymes is enhanced, accelerating the formation of dental caries. Correspondingly, reduced levels of IgG in smokers’ saliva can diminish the body’s ability to respond to infection and increase the risk of developing autoimmunity (63).

The immune system serves to protect the body from external pathogens. Long-term exposure to tobacco smoke may impair immune function, induce immunodeficiency, and alter the oral flora, leading to tooth decay. It has been suggested that a compromised immune system, in addition to promoting colonization of *Streptococcus mutans*, also lowers vitamin C levels in children exposed to smoke. Reduced vitamin C levels are strongly associated with the proliferation of cariogenic bacteria, aligning with findings from Väänänen’s research (64, 65). Therefore, exposure to tobacco smoke may indirectly promotes the growth and colonization of bacteria associated with dental caries by impairing human immune function and reducing vitamin C levels in children, or by directly altering the oral microflora.

#### 2.2.2 Impaired immune function

Tobacco smoke may affect the mediators of the immune system, thereby increasing susceptibility to caries. For instance, nicotine inhibits the expression of signaling molecules involved in dendritic cell maturation, T-cell activation, and cell migration, and reduces the immunogenicity of dendritic cells at the signaling pathway level, displaying immunosuppressive properties (66). Researchers suggest that tobacco smoke can damage proteins and DNA, which adversely affects the development of dentin and tooth enamel, rendering the teeth more vulnerable to oral pathogens and leading to caries. Additionally, tobacco smoke may compromise mediators involved in the immune system (67). Exposure to cigarette smoke during pregnancy can negatively impact the immune system by influencing toll-like receptors and altering the function of fetal T-helper, Th-1, and Th-2 cells, resulting in increased susceptibility to caries post-birth. Studies by Aderonke and Julihn et al. have confirmed the link between prenatal smoking and dental caries in offspring and have indicated that smoking during the first trimester of pregnancy poses a higher risk of dental caries in offspring than smoking in other trimesters, likely due to the critical period for tooth formation during the first trimester (68). These findings are consistent with research by Keiko, T., which shows that maternal smoking during the first trimester is associated with a significant 40% increase in the risk of dental caries in children. Moreover, it demonstrates that maternal smoking during pregnancy, combined with postpartum exposure to tobacco smoke, has a compounding effect on dental caries in children (69). Maternal smoking has been found to be more strongly associated with tooth decay in children than in fathers, but further evidence is needed, for example, to explore whether disturbances in the fetal immune response affect early microbial colonization of tooth surfaces.

#### 2.2.3 Epigenetic effects

Research indicates that ETS may have epigenetic effects on genes involved in immune function, thereby increasing resistance to bacteria (45). Epigenetic changes, such as alterations in gene function, are often caused by methylation in the gene promoter region, a process dependent on methyl groups primarily sourced from dietary folate. However, maternal smoking can impair the transport of folic acid across the placenta in a dose-dependent manner, even minimal exposure to tobacco smoke can affect ascorbate levels in children. In addition, as for the mechanism by which epigenetic changes affect caries, we speculate that on the one hand, it may affect the occurrence and progression of caries without changing the DNA sequence by affecting the expression of genes associated with caries. On the other hand, early life is the most sensitive period for exposure to environmental factors, which often lead to varying degrees of enamel development defects, and epigenetic mechanisms may play a role in this process, affecting the normal development of tooth enamel and leading to poor enamel mineralization. Finally, epigenetic mechanisms may affect the function of odontogenic stem cells, which in turn affects the development of caries.

Tobacco smoke not only disrupts innate immune mechanisms, but also some adaptive immune mechanisms. After quitting smoking, its effects on the innate immune response disappear quickly, while the effects on adaptive immunity persist for a long time, lasting 10 to 15 years. This suggests that smoking may have long-term effects on the immune response, which is related to epigenetic memory.

### 2.3 Saliva

Dental caries, a prevalent multifactorial disease, affects a significant portion of the global population. Saliva, which continuously bathes the teeth, plays a crucial role in both the development and progression of dental caries. It is considered a key factor in controlling the cariogenic pathway, influencing both the speed and direction of caries formation. Although findings vary, the existing literature generally supports a higher prevalence and/or incidence of caries among individuals with low saliva flow rate, compromised buffering capacity, and early colonization or high levels of mutans streptococci in saliva (70). However, evidence regarding the association between dental caries and other saliva parameters, such as other potential cariogenic species (*Lactobacillus* spp., *Streptococcus sanguis* group, *Streptococcus salivarius*, *Actinomyces* spp., and *Candida albicans*), diversity of saliva microbiomes, and inorganic and organic constituents (electrolytes, immunoglobulins, other proteins, and peptides), as well as certain functional properties (sugar clearance rate, etc.) remains weak and/or inconsistent. Given the complex interactions between salivary components and functions, it is crucial to consider saliva as a whole to fully understand its impact on teeth. Tobacco smoke, mainly through harmful components like nicotine, induces a series of changes in the body that can reduce the buffering capacity of saliva, alter its chemical agents and bacterial composition, and thus promote the formation of a caries-prone environment (14). Furthermore, it has been found that nicotine stimulates the release of neurotransmitters (71) at synapse-associated nerve endings, activates Ca^2+^ in rat parotid vesicles, and induces an increase in [Ca^2+^] in parotid acinar cells. Correspondingly, children with multiple caries exhibit an increased concentration of calcium ions in their saliva. This shows that nicotine in tobacco smoke can promote the development of dental caries by increasing the concentration of calcium ions in saliva.

#### 2.3.1 Salivary flow rate

Numerous studies have consistently shown that long-term smoking significantly impacts the salivary flow rate (SFR) and the development of dental caries (44). SFR is considered a crucial parameter for the inhibitory activity of salivary functions against caries. It has been found that smokers have a significantly lower average SFR compared to non-smokers (72), a finding supported by Thomson et al. Further research has revealed that the inhibitory effect of smoking on SFR is delayed, with a noticeable decrease observed only 1 h after smoking (60).

There are two main mechanisms through which smoking affects SFR and contributes to the increased risk of dental caries among smokers. Firstly, the decrease in SFR weakens the flushing effect of saliva, allowing for the accumulation of food residues, creating a favorable environment for the growth of microorganisms, and potentially increasing the incidence of dental caries (73, 74).

Secondly, long-term smoking can lead to the development of tolerance and reduced taste perception, resulting in weakened salivary reflex and ultimately reducing SFR (75, 76). Presently, several hypotheses have been proposed to explain how smoking reduces the sensitivity of taste receptors, including significant changes in the shape, size, and vascularization of fungiform papillary structures (77), a decrease in the number of taste cells (78), and substantial alterations in the structure of salivary glands (79). In the future, it is expected that the mechanism of smoking reducing taste receptor sensitivity will be further explained through the improvement of relevant research content.

#### 2.3.2 Saliva buffering capacity

Research has shown that suppressing the buffering capacity of saliva results in lower saliva pH and higher populations of acidophilic bacteria in the mouth. Consequently, this elevates the risk of dental caries. Smoking diminishes salivary buffering capability by reducing the SFR, which subsequently lowers bicarbonate secretion (73). Bicarbonate, a crucial element of the salivary buffering system, operates alongside phosphate and protein buffers (80). As a result, smoking leads to a decrease in saliva pH, which promotes the development of caries.

#### 2.3.3 sIgA

Saliva plays a vital role in oral health and homeostasis through its physicochemical properties and the biological activity of its components, including salivary immunoglobulin A (sIgA). sIgA is a critical defense factor against oral diseases, inhibiting the adhesion of microorganisms to tooth surfaces and oral epithelial cells, thus decreasing bacterial colonization and the risk of dental caries. Lowered sIgA concentrations are associated with an increased risk of dental caries in both children and adults (81, 82). Smoking significantly reduces sIgA levels in saliva. Studies indicate that sIgA levels in the saliva of caries patients are significantly reduced compared to healthy controls. Salivary sIgA has been proposed as an alternative indicator for identifying individuals at risk of dental caries, underscoring its potential as a protective factor (83).

Smoking suppresses immunoglobulin secretion, resulting in decreased sIgA levels in saliva. Observational studies have shown that smokers consistently exhibit lower average sIgA levels than non-smokers (84). Furthermore, smokers with dental caries have lower sIgA levels in their saliva compared to their counterparts without caries, corroborating earlier research findings (82).

Another mechanism is that smoking exhibits dose-dependent immunosuppressive effects. Nicotine exposure from cigarette smoke suppresses immune function, leading to reduced salivary sIgA levels and an elevated risk of dental caries. Cotinine, a biotransformation product of nicotine, is detectable in smokers’ saliva, with studies demonstrating a significant inverse correlation between cotinine levels and sIgA (85).

**Figure 3:**
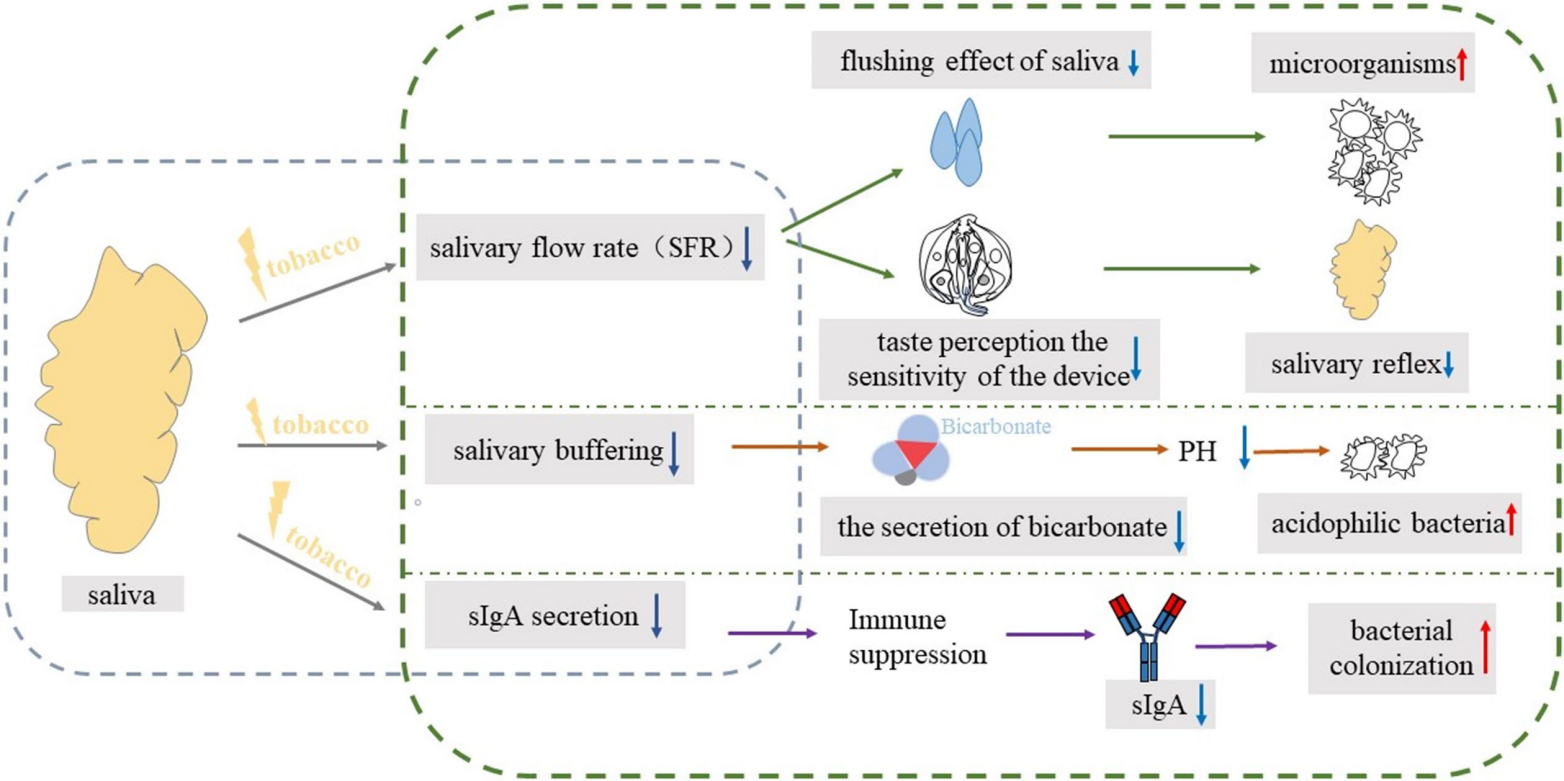
The blue box on the left represents the impact of tobacco exposure on saliva, while the green box on the right details the specific cariogenic mechanisms. The blue downward arrow signifies a decrease, and the red upward arrow indicates an increase.

In conclusion, long-term exposure to environmental tobacco smoke may lead to dysbiosis of the oral flora, making cariogenic bacteria (e.g., *Streptococcus mutans*) the dominant flora. These bacteria metabolize sugars to produce a large amount of acidic substances that reduce the pH of oral saliva, thereby eroding tooth enamel and forming cavities; It may also directly affect the secretion and composition of saliva (Figure 3), reduce the concentration of immunoglobulins (such as SIgA) in saliva, and weaken the antibacterial and buffering capacity of saliva, which makes the oral cavity more susceptible to cariogenic bacteria and promotes the further development of dysbiosis.

### 2.4 Oxidative stress

An imbalance between free radicals and antioxidants defines oxidative stress (OS) (86). Recent studies demonstrate that PM exposure influences oral health by increasing oxidative stress. In children with active caries, variations in salivary biomarkers—specifically decreased total oxidative status (TOS) and increased total antioxidant status (TAS)—suggest that active caries may shift the oxidative/antioxidant balance toward a predominantly antioxidant state after treatment. While environmental particulate matter-induced oxidative stress is thought to be one of the main mechanisms, antioxidant supplementation has not been shown to significantly improve health, suggesting that there may be other, more important mechanisms (87, 88).

Tobacco smoke, rich in potent oxidants such as oxygen radicals and volatile aldehydes, can damage biomolecules like proteins and enzymes, leading to various physiological disturbances (89, 90). Exposure to tobacco smoke causes oxidative/antioxidative imbalance, marked by inflammatory infiltration with abundant neutrophils, increased protease secretion, and a surge in oxidative intermediates. Martins et al. (91) noted that tobacco smoke exposure induces oxidative stress, leading to an increase in reactive oxygen species (ROS), with the tooth decay mechanism possibly involving infiltration by inflammatory cells such as monocytes, macrophages, and neutrophils. This infiltration intensifies oral inflammation via oxidative stress, disrupting oral cavity function and potentially causing apoptosis, thereby damaging the hard tissues of the teeth. Studies have linked oxidative stress with caries development, noting that high glucose intake increases oxidative stress in the hypothalamus. This process, when disrupted, can halt or reverse the flow of dentin, allowing acidogenic bacteria such as Lactobacillus to adhere to tooth surfaces, demineralize enamel, and activate dentin. Acid erosion then triggers the body’s matrix metalloproteinases to degrade dentin.

Tobacco smoke induces apoptosis through oxidative stress, which is a crucial factor in dental damage. Apoptosis is integral to cell growth and response to external stimuli, with oxidative stress playing a central role in apoptosis initiation (92). The NF-E2-related factor 2 (Nrf2) signaling pathway acts as a critical regulator of cellular antioxidative responses, controlling oxidative stress-induced enzymes and bolstering cellular defense against peroxidative stress. In conditions of oxidative damage, Nrf2 binds to the antioxidant response element (ARE), activating genes for phase II detoxification enzymes such as heme oxygenase-1 (HO-1) and NAD(P)H dehydrogenase quinone 1 (NQO1), thus enhancing the body’s resistance to oxidative stress (93). Prolonged exposure to tobacco smoke may disrupt the Nrf2 pathway, impair the antioxidant system, unbalance oxidative states, and promote apoptosis by inducing oxidative stress. For the Nrf2 pathway, some studies have mentioned the use of natural compounds as potential Nrf2 activators for the development of topical drugs for the treatment of caries. Therefore, Nrf2-targeted therapy has the potential to be a potential prevention strategy.

In one study, biomarkers of oxidative stress and related parameters in saliva were associated with dental caries, with increased antioxidant biomarkers (TAC and SOD) and total protein concentrations in saliva in children with caries (94), while indicators of oxidative damage to saliva (MDA) and parameters such as saliva flow rate, pH, buffering capacity, and calcium showed lower values. At the same time, in a study evaluating dental caries in diesel generator workers, salivary protein carbonyl compounds, a stable marker of ROS protein oxidation, were positively correlated with dental caries (95). Protein carbonyls are acknowledged as a stable end product of protein oxidation and are the prevalent biomarkers for oxidative protein damage (96).

### 2.5 Oxygen deprivation

The carbon monoxide in tobacco smoke can combine with hemoglobin to form carboxyhemoglobin, which impairs the oxygen-carrying capacity of red blood cells, causing tissue hypoxia and causing harm to tissue cells. Research indicates that caries recurrence in affected teeth is linked to hypoxia-dependent activation of innate antimicrobial immunity, characterized by elevated levels of hypoxia-inducible factor-1*α* (HIF-1α) and a notable increase in α-defensins 1–3 in gingival fluid (97). An initial HIF-1α level of 98 pg./mL is associated with a high risk of caries recurrence within 12 months post-treatment, showing diagnostic sensitivity of 88.4% and specificity of 78.1%, with a fourfold increase in risk (*p* < 0.0001). By assessing HIF-1α levels in gingival fluid, it is possible to identify patients at high risk of caries recurrence, who may benefit from enhanced dental monitoring and personalized preventive measures (98). At the same time, hypoxia also significantly affects dental pulp. Pan et al. examined the apoptotic responses of human dental pulp cells (HDPCs) under various oxygen and serum levels to simulate different ischemia degrees. They explored how lysophosphatidic acid (LPA) could mitigate ischemia-induced apoptosis and investigated the underlying mechanisms. The study revealed that LPA effectively rescues HDPCs from apoptosis by modulating Bax and Bcl-2 proteins and activating phosphorylated FAK and ERK, highlighting its potential as a biological therapy for chronic pulpal inflammatory diseases (99).

The effect of hypoxia on caries has been accompanied by the whole process of caries occurrence and development. As carious infections progress toward the pulp-dentin interface, alterations in microflora composition occur. These are marked by a decrease in Gram-positive aerobic bacteria and an increase in Gram-negative anaerobic bacteria (100). Consequently, the environment within deep carious lesions becomes more anaerobic, fostering more complex polymicrobial infections with increased bacterial diversity (101). This enhanced complexity may exacerbate the severity of caries.

## 3 Coal-fired particles

Industrial dust, including coal-fired particles, is a significant source of PM_2.5_. Coal-fired particles refers to the impure carbon particles released into the environment during diesel engine combustion, which is one of the main factors causing air pollution. It is worth noting that coal-fired particles contains carcinogenic polycyclic aromatic hydrocarbons, making it a risk factor for caries. Clinical observations have reported that tooth-brushing with raw charcoal, a component of soot, can have detrimental effects, such as an increased risk of caries (102). In addition, an experiment conducted on white rats showed that exposure to industrial environmental factors, including coal-burning particles, led to a higher incidence of dental caries. The mechanism is that exposure to coal-fired particles leads to changes in metabolic processes, including those leading to disruption of the salivary enzyme system, leading to an increased prevalence of dental caries (103). Since particulate matter from coal combustion mainly comes from coal combustion, industrial emissions, and vehicle exhaust, policymakers can set up air quality regulations to restrain the sources of coal pollution, which not only improves air quality, but also provides indirect protection for reducing the risk of caries.

### 3.1 Chemical element

Coal burning particulate matter can potentially impact the occurrence of dental caries by influencing the composition of essential chemical elements in tooth enamel and dentin. Airborne dust, nitrogen oxides, sulfur-containing gasses and carbon dioxide can cause changes in the macro-and microelemental composition of saliva and tooth tissue, affecting the crystal structure in tooth enamel, which may be one of the causes of tooth decay (104). There has also been epidemiological evidence that silica in coal-fired particles may affect caries, and a study of underground coal miners showed a high prevalence of dental caries due to their long-term exposure to coal dust and silica particles (105). In addition, among stone miners in Jodhpur, Rajasthan, India, workers exposed to dust had a higher incidence of dental caries (94), which is consistent with the findings of Petersen and Henmar (106).

While there is no direct causal relationship between coal particulate matter and caries, it can be speculated that coal particulate matter may indirectly affect oral health by affecting overall health. For example, systemic inflammation caused by coal-fired particulate matter may affect the body’s resistance to oral infections, or certain components of coal-fired particulate matter may affect the balance of the oral microbiome after dietary intake. However, these hypotheses require further scientific research to validate.

### 3.2 The immune system

Research has indicated that prolonged exposure to coal smoke can weaken the immune system (107, 108). Air pollution can affect both the non-specific immunity and humoral immunity of the human body. When the immune system is affected, it promotes the growth of cariogenic bacteria in the oral cavity and reduces the level of salivary lysosomal coal, leading to an imbalance in the oral microbiota and the development of dental caries. Therefore, a weakened immune system may also be a potential cause of sooty-induced caries, but the specific mechanism of action is still unclear and more research is needed.

### 3.3 Oxidative stress

Oxidative stress (OS) is a condition characterized by an imbalance between oxidative and antioxidant effects in the body. This imbalance leads to the inflammatory infiltration of numerous neutrophils, increased secretion of proteases, and the production of oxidative intermediates. The researchers have indicated that exposure to coal-fired particles induces oxidative stress and generates excessive reactive oxygen species (ROS). The mechanism by which this leads to dental caries may involve the infiltration of inflammatory cells such as monocytes, macrophages, and neutrophils, exacerbating oral inflammation through oxidative stress. This, in turn, results in partial functional abnormalities or even cell apoptosis in the oral cavity, ultimately causing damage to the hard tissue of teeth (16).

The protein carbonyl group is considered a stable end product of protein oxidation caused by reactive oxygen species (ROS) and is widely used as a biomarker for oxidative protein damage (96). In one study, a group of diesel generator workers were evaluated on the experience of dental caries and the relationship between salivary oxidative stress biomarkers, protein carbonyls, and dental caries. The study found a higher incidence of caries among workers who worked longer hours, and the experience of caries increased as well. Additionally, the study revealed a positive correlation between the protein carbonyl group in saliva and caries (95). To a certain extent, part of the mechanism by which coal-fired particles lead to caries by affecting oxidative stress levels has been revealed.

## 4 Metal particles

**Figure 4:**
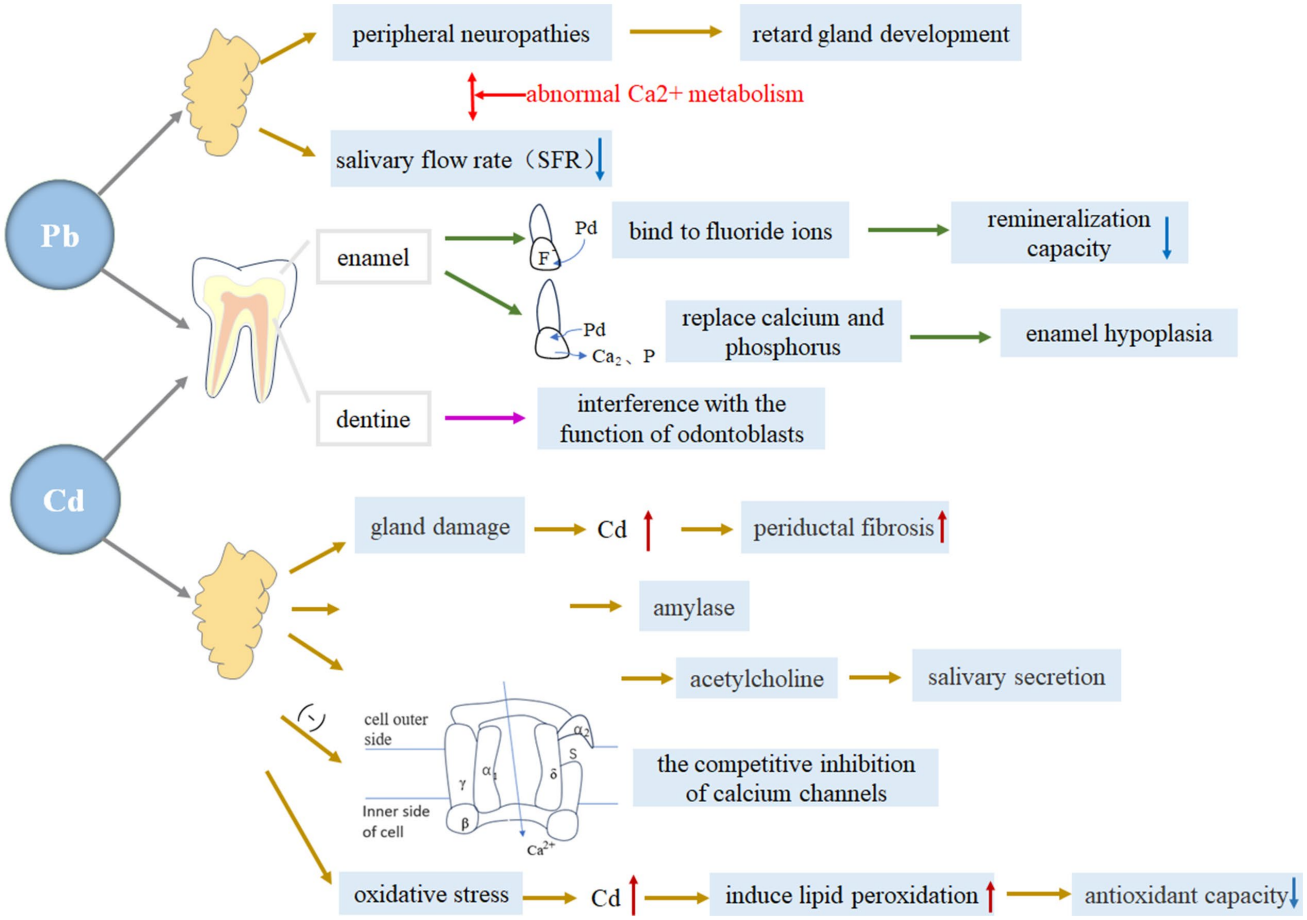
The specific mechanism of lead and cadmium on tooth enamel, dentin and salivary glands. Blue downward arrows indicate decreased levels; red upward arrows indicate increased levels.

Lead (Pb) and cadmium (Cd) are significant environmental pollutants that can have detrimental effects on the body. These metals can accumulate in various organs and impact the growth and development of children, potentially leading to metal poisoning. The adverse effects of lead on oral tissues have been recognized for centuries. Ingesting certain metals, especially lead, has long been known to pose a major health hazard. Studies conducted over the past two decades have demonstrated a relationship between blood lead levels and dental caries, with environmental lead exposure associated with a higher prevalence of tooth decay in the American population (109, 110). Pradeep Kumar KN et al. analyzed lead levels and caries in tooth enamel and saliva of 5-year-old children living in areas with high lead content, further confirming the caries-causing potential of lead (111). There are a number of mechanisms that explain how lead and cadmium contribute to the development of dental caries, among which increasing susceptibility to dental caries by affecting tooth enamel, dentin, and salivary glands has been studied more (Figure 4).

### 4.1 Tooth enamel

Numerous studies (112–114) have consistently demonstrated the detrimental effects of lead and cadmium on the formation of tooth enamel and dentin, rendering teeth more vulnerable to caries.

The mechanism behind this correlation is believed to involve enamel demineralization. Specifically, an increase in enamel lead levels during the normal demineralization and remineralization cycle can lead to the development of enamel defects or alterations in the enamel’s physicochemical properties, ultimately contributing to an increased caries rate (115, 116). Animal studies have provided evidence of enamel defect formation, showing that lead exposure leads to decreased hardness in less-mature enamel (117). Furthermore, a relationship has been observed between lead and changes in the physicochemical behavior of enamel, whereby lead can bind to fluoride ions present in saliva and plaque, reducing the ability of fluoride to remineralize enamel following an acid challenge (118). This, in turn, makes the enamel more susceptible to demineralization (119).

The second mechanism of lead on caries is its direct interaction with bone minerals. Lead replaces calcium and phosphorus in the crystal lattice, resulting in underdeveloped tooth enamel. A study has shown that lead may have a direct impact on the mineral phase of calcifying tissues (120). It has been suggested that lead initially adsorbs to hydroxyapatite crystals and later integrates into the structure (121). Previous research has demonstrated the widespread dispersion of lead when Pb hydroxyapatite is synthetically formed (122). It has been observed that the replacement of calcium by lead is a slow process, but lead can be quickly incorporated into apatite in a dynamic mineralizing system, increased susceptibility to dental caries (123). Previous evidence has reported that this mechanism can induce hypercalcemia and hyperphosphatemia (124, 125). Furthermore, it may contribute to an increased incidence of enamel hypoplasia in children and animals exposed to elevated levels of lead (126).

### 4.2 Tooth dentine

Lead may influence dentine formation through its impact on odontoblast function (127). Previous studies have demonstrated that lead intoxication can directly and indirectly affect various aspects of bone cell function (128). Research has indicated that lead alters bone cell function by causing changes in 1,2,5 dehydroxyvitamin D3 levels (129). Furthermore, it has been documented that lead can disrupt cellular response to hormonal regulation and impair the synthesis of collagen or bone sialoproteins. Additionally, lead may directly affect or substitute for calcium in the active sites of the calcium and cAMP messenger systems (119). Currently, there is a lack of research investigating the impact of cadmium on dentin.

### 4.3 Salivary glands

The main function of the salivary gland is to secrete saliva, which not only has a great effect on digestion, but also is closely related to taste, language, swallowing and other functions, and its role involves oral hygiene, mucosal protection and caries prevention. When salivary gland dysfunction occurs, it can lead to a decrease in saliva production, which increases the risk of tooth decay. Heavy metals such as lead and cadmium can not only accumulate through the salivary glands and aggravate oxidative stress, but also have a series of adverse effects on human metabolism, including a decrease in saliva secretion, which indirectly causes the occurrence and development of caries. In addition, studies (130) have shown that the composition of the salivary microbiome changes after exposure to environmental toxicants (e.g., heavy metals), leading to oral caries.

#### 4.3.1 Lead exposure effect

The presence of lead in the environment can negatively affect the development and function of the salivary glands. And it produces a bacterial inhibition effect through saliva (131).

Lead has been found to have an impact on the nervous system, causing peripheral neuropathies such as slower maturation of synaptic density (132), reduction in conduction rates (133, 134), and depression of pre-synaptic release of acetylcholine in the superior cervical ganglion (135, 136). Studies have shown that both sympathetic (137–139) and parasympathetic (140) denervation can retard gland development. At the same time, it has been shown that there is a correlation between lead exposure and inhibition of calcium uptake, and that lead exposure has a certain inhibitory effect on neural responses, and the response to the oral cavity can lead to salivary gland developmental retardation, especially in the perinatal period, which is more likely to have long-term effects on saliva function, because it interferes with the normal interaction between the autonomic nervous system and the salivary glands during development (132).

Another factor to consider is the direct impact of lead on gland tissue, which can inhibit saliva formation. Although this phenomenon has not been studied in humans, studies on rats have shown that lead administration significantly reduces stimulated salivary flow rates (141, 142). Lead disrupts normal Ca2 + metabolism, leading to acute alterations in cell function (119, 143, 144). This interference with Ca2 + metabolism has severe consequences on salivary gland function. Therefore, it can be concluded that lead’s interaction with Ca2 + metabolism is one of the most likely mechanisms through which it acutely interferes with saliva formation.

#### 4.3.2 Cadmium exposure effect

There is a limited amount of research on the relationship between environmental cadmium exposure and caries. A study conducted by Manish Arora et al. revealed a potential association between environmental cadmium exposure and a higher risk of caries in primary teeth among children. The mechanism by which cadmium can increase the incidence of dental caries has been investigated (145). Cadmium exposure, then, may lead to an increased prevalence of dental caries.

The first mechanism is cadmium-induced destruction of the salivary ducts. Studies have shown that when cadmium is administered subcutaneously to rats, it leads to histologic signs of tubular and acinar damage in salivary glands. The severity of this damage correlates with the degree of gland dysfunction (146). Additionally, an experiment conducted on rats found that periductal fibrosis increased in the submandibular glands with higher doses of cadmium (147). This reduces saliva secretion, which affects the development of caries.

Furthermore, it has been observed that cadmium exposure has a detrimental effect on salivary secretions. Specifically, it decreases the concentration of amylase, which is a crucial digestive enzyme found in parotid gland saliva (146). Decreased amylase concentrations may affect the efficiency of the removal of food debris in the oral cavity and the balance of the oral microbial community, which may increase the risk of caries.

The third aspect is related to synaptic transmission. Previous studies have demonstrated that cadmium inhibits the release of acetylcholine and disrupts parasympathetic impulses, which play a significant role in regulating salivary secretions (148). Additionally, a previous study has revealed that both evoked and spontaneously released quanta of transmitter likely act on the same population of postsynaptic receptors in submandibular ganglion cells (149).

The fourth aspect is the competitive inhibition of calcium channels by cadmium. The accumulation of cadmium was higher under low calcium conditions, and increasing the calcium concentration could reduce the accumulation of cadmium. This suggests that calcium may reduce cadmium uptake by competing with cadmium for absorption sites. A previous study has documented that an increase in calcium secretion caused by cadmium leads to a decrease in salivary flow rate, total protein concentration, and amylase activity in saliva (150).

The fifth mechanism is the induction of oxidative stress in salivary glands. Cadmium has been known to cause toxic actions in various organs of the body through oxidative stress (151). Studies have shown that acute administration of cadmium (10 mg/kg) leads to an increase in lipid peroxidation by-products and a decrease in total thiols and total antioxidant power of saliva, resulting in significant oxidative stress. Furthermore，in experiments conducted on cadmium-treated rats, it was observed that there was a significant increase in lipid peroxidation in submandibular saliva and a significant decrease in total thiol levels in saliva (152). Therefore, it can be concluded that cadmium induces lipid peroxidation and reduces the levels of total thiol groups and antioxidant power in submandibular saliva. Reduced total sulfhydryl levels may imply a decrease in antioxidant capacity, which may make the oral environment more susceptible to cariogenic bacteria and may also lead to the accumulation of free radicals in the oral cavity that damage tooth structure and thus increase the risk of tooth decay.

## 5 Summay

In this review, the three most important components of environmental particulate matter, tobacco smoke, coal-burning particles, and heavy metal particles, were selected and discussed the mechanisms by which they may affect the development of dental caries (Table 1). The research on particulate matter mainly focuses on its effects on oral microecology, saliva, immune system and oxidative stress, and preliminarily explores the process of their influence on the occurrence and development of caries, which has certain explanatory significance for the mechanism of interaction between these factors. This study highlights the critical role of oxidative stress in the development of oral caries. Long-term exposure to tobacco smoke and coal-fired particles lead to an imbalance between oxidative and antioxidant capacities in the body, triggering extensive infiltration of inflammatory cells, which exacerbates oral inflammation and affects the hard tissues of the teeth, indirectly facilitating caries development. Activation of the hypothalamus also increases the attachment of cariogenic bacteria to the tooth surface, promoting the onset and progression of dental caries. The Nrf2 signaling pathway, a pivotal nuclear transcription factor in cellular antioxidative response, is hypothesized to be impaired by environmental particulate matter, weakening the body’s oxidative stress defense and leading to an imbalance between oxidative and antioxidant activities. This results in oxidative stress, induces apoptosis, and ultimately contributes to the development of caries.

**Table 1:** A summary table of the key mechanisms for different PM types.

Type	Related mechanisms	Key role	Cite
Environmental tobacco smoke	Imbalance of microflora	Nicotine in tobacco smoke can upregulate the expression of glucan-binding protein (GBP) and GTF genes in Streptococcus plankton, and downregulate their expression in biofilm communities, promoting bacterial adhesion and accumulation on the tooth surface and subsequent acid production, leading to tooth demineralization and caries formation.	(38)
		Vertical transmission: Transmission of *Streptococcus mutans* by smoking mothers to her children can be an important factor in tooth decay in children	(45)
		Heavy metals such as arsenic, lead, and cadmium present in smoke can accumulate in the oral cavity, disrupting the acid–base balance and accelerating the activity of pathogenic bacteria, promoting the determination of lactobacilli values	(47)
		The nicotine in tobacco can enhance the competitive advantage of *Streptococcus mutans* over Streptococcus haemoides and promote the development of dental caries.	(40)
	Immune system	Tobacco smoke may impair immune function, induce immunodeficiency, lower vitamin C levels in children, and promote the proliferation of bacteria associated with dental caries.	(64)
		Tobacco smoke can damage the mediators involved in the immune system, leading to increased susceptibility to tooth decay after birth	(67)
	Saliva	Reduces saliva flow rate, reduces the ability of saliva to flush, and promotes microbial attachment.	(74)
		Inhibits the buffering capacity of saliva, resulting in a decrease in PH and promoting the development of dental caries.	(73)
		Inhibition of immunoglobulin secretion results in a decrease in sIgA levels in saliva, increasing the risk of bacterial stabilization.	(81, 84)
	Oxidative stress	Damage teeth by disrupting the Nrf2 pathway, impairing the antioxidant system, impairing the oxidative state, and promoting apoptosis by inducing oxidative stress.	(92, 93)
	Anoxia	Long-term exposure to tobacco smoke leads to ischemia and hypoxia of pulp cells, resulting in a decrease in gram-positive aerobic bacteria and an increase in gram-negative anaerobes, promoting more complex microbial infections, increasing bacterial diversity, and exacerbating the severity of dental caries.	(101)
Coal-fired particles	Affects the host	Exposure to coal-fired particles can lead to changes in metabolic processes, including those leading to disruption of the salivary enzyme system, leading to an increased prevalence of dental caries.	(103)
Metal particles	Demineralization of tooth enamel	An increase in the level of lead in enamel can lead to the development of enamel defects or alterations in the physicochemical properties of enamel, ultimately leading to an increase in the rate of tooth decay.	(115, 116)
	Enamel hypoplasia	Lead displaces calcium and phosphorus in the crystal lattice, resulting in underdeveloped tooth enamel.	(120)
	Decreased saliva production	Lead disrupts normal Ca2 + metabolism, leading to acute alterations in cellular function that acutely interfere with saliva formation.	(143)
	Destruction of the salivary ducts	Exposure to cadmium aggravates periductal fibrosis of the salivary glands and reduces saliva secretion, which affects the development of dental caries.	(147)
	Lowers salivary amylase	Decreased amylase concentrations may affect the efficiency of removing food debris from the mouth and the balance of the oral microflora, increasing the risk of dental caries.	(146)
	Oxidative stress	Cadmium induces lipid peroxidation and decreases the level of total sulfhydryl groups and antioxidant capacity in saliva, leading to increased host susceptibility or damage to teeth, increasing the risk of dental caries.	(152)

Many studies report an association between PM and caries, but few establish a causal link. Most of these studies are cross-sectional studies, and although this design can provide some preliminary information, it is difficult to reveal the causal relationship and dynamic mechanism between the two due to the lack of a time dimension, and there are also great challenges in controlling confounders. In order to establish a causal relationship between environmental particulate matter exposure and caries, it is necessary to control for potential confounding variables (such as socioeconomic, educational, and behavioral factors) in the future, and to make specific and quantitative measurements of particulate matter exposure through prospective longitudinal studies and controlled trials, so as to more accurately assess the relationship between exposure levels and health effects, and reduce uncertainties caused by inaccurate exposure assessments; At the same time, the heterogeneity of the impact of particulate matter sources and composition differences on caries is revealed, so as to provide a scientific basis for the development of more effective pollution control strategies. In addition, more studies should try to establish the dose–response relationship between particulate matter and caries, and study the toxic effects of various particulate matter, so as to provide clues for further exploration of the toxicity mechanism of particulate matter.

This study illustrates the influence of environmental particulate matter on the occurrence and development of caries, and is expected to prevent the occurrence of caries by adjusting specific influence mechanisms, hinder its further development, and avoid the aggravation of the disease. At the same time, this study has certain guiding significance for the development of interventions for the prevention and treatment of caries. The correlation between environmental particulate matter and caries illustrated in this study has implications for researchers, policymakers, and public health workers. Researchers can focus their research on exploring the specific mechanisms between environmental particulate matter and caries, combining experimental studies, epidemiological investigations, etc.; Public health professionals can incorporate the control of important components in environmental particulate matter into oral health promotion programs as an important strategy to prevent caries and evaluate the effectiveness of interventions; Policymakers can introduce special protection policies for sensitive groups (e.g., children, the older adult) to promote the use of clean energy and reduce environmental pollution. In addition, this study can carry out more accurate individual treatment, and implement key prevention and treatment for high-risk groups, so as to promote the overall improvement of the effective prevention and treatment of caries.
